# Early Rupture of the Membranes in Midwifery

**Published:** 1878-02

**Authors:** William Goodell


### Early Rupture of the Membranes in Midwifery.

In your issue of December 22d, at page 497, reference is made to an analysis given by an English surgeon, Mr. Plaister, of 800 consecutive midwifery cases. In it he warmly urges the early rupture of the membranes, and says: "I have never found any ill-effects from rupturing the membranes when the os is the size of a shilling, but find that the child's head forms a better wedge than the bag of liquor amnii. I cannot recall any instance where there has been any retarding of labor through this procedure."

Now, although this advice has the great weight of your own sanction, I cannot allow it to pass unchallenged. While granting, with you, that there is no surer way of shortening the first stage of labor than by breaking the bag of waters, I hold that the practice is a mischievous one—one threatening the future health and happiness of the woman—and that, therefore, it should never be adopted save for urgent reasons.

Perhaps, next to abortion, the most common cause of prolonged lochial discharges, of a bad getting up, of pelvic and uterine disorders, of stubborn sub-involution, is the laceration of the cervix. Now, for obvious reasons, nothing is more likely to produce this lesion than the early escape of the waters, especially in a primipara

Hence it is that many a life-long invalidism dates from the first labor.

Having myself made the mistake of prematurely breaking the membranes, and having to deplore the consequences, I am the better enabled to warn others, and to denounce this practice as meddlesome midwifery. True it is, that some few women do not experience any bad effects from a laceration of the cervix, but the vast majority do. From the attrition and irritation of the now unshielded delicate lining membrane of the cervical canal, the womb becomes hypertrophied, and the pouting mucous membrane looks so raw and angry that it is constantly being mistaken for "ulceration," or for cancer, and is accordingly mistreated. So common, indeed, is this accident of child-birth, for accident it certainly is, since nature never intended it, that, out of every five women who apply to the Dispensary for the Diseases of Women at the Hospital of the University of Pennsylvania, I find one who is suffering from its effect. Repeatedly have I operated for it, in one week three times, one of the cases being, to my shame, a woman whom I myself had previously delivered.

In view of these facts, can I begin the new year better than by urging on the readers of your valued journal the adoption of the general rule, of not breaking the bag of waters in primiparæ until the os uteri has *fully* dilated, and in multiparæ until fair dilatation has taken place, a dilatation certainly greater than "the size of a shilling?" Very faithfully yours,

William Goodell, M. D.

*Medical and Surgical Journal.*

